# RAPAMYCIN INCREASES LENGTH AND MECHANOSENSORY FUNCTION OF PRIMARY CILIA IN RENAL EPITHELIAL AND VASCULAR ENDOTHELIAL CELLS

**Published:** 2016-12

**Authors:** Rinzhin T. Sherpa, Kimberly F. Atkinson, Viviana P. Ferreira, Surya M. Nauli

**Affiliations:** 1 Department of Biomedical & Pharmaceutical Sciences, Chapman University, Irvine, CA; 2 Department of Medical Microbiology and Immunology, University of Toledo, Toledo, OH

**Keywords:** cilium, kidney, mechanosensation, shear-stress, vascular

## Abstract

Primary cilia arebiophysically-sensitive organelles responsible for sensing fluid-flow and transducing this stimulus into intracellular responses. Previous studies have shown that the primary cilia mediate flow-induced calcium influx, and sensitivity of cilia function to flow is correlated to cilia length. Cells with abnormal cilia length or function can lead to a host of diseases that are collectively termed as ciliopathies. Rapamycin, a potent inhibitor of mTOR (mammalian target of rapamycin), has been demonstrated to be a potential pharmacological agent against the aberrant mTOR signaling seen in ciliopathies such as polycystic kidney disease (PKD) and tuberous sclerosis complex (TSC). Here we look at the effects of rapamycin on ciliary length and function for the first time. Compared to controls, primary cilia in rapamycin-treated porcine renal epithelial and mouse vascular endothelial cells showed a significant increase in length. Graded increases in fluid-shear stress further indicates that rapamycin enhances cilia sensitivity to fluid flow. Treatment with rapamycin led to G0 arrest in porcine epithelial cells while no significant change in cell cycle were observed in rapamycin-treated mouse epithelial or endothelial cells, indicating a species-specific effect of rapamycin. Given the previousin vitro and in vivo studies establishing rapamycin as a potential therapeutic agent for ciliopathies, such as PKD and TSC, our studies show that rapamycin enhances ciliary function and sensitivity to fluid flow. The results of our studies suggest a potential ciliotherapeutic effect of rapamycin.

## Introduction

The primary cilium is a solitary cellular organelle that protrudes from the apical cell membrane. Studies on cilia-dependent mechanosenstation have shown that the primary cilium acts as a transducer of fluid-shear stress into intracellular signaling (Masyuk et al., 2006, Nauli et al., 2003, Nauli et al., 2008). Along with its mechanosensory function, the primary cilium houses a variety of receptors, ion channels, transporter proteins, and other protein complexes involved in signal transductions, such as the Hedgehog(Huang and Schier, 2009),Wnt (Corbit et al., 2005), planar cell polarity(Ross et al., 2005) and platelet-derived growth factor(Schneider et al., 2005)pathways.

Abnormalities in cilia structure or function lead to a spectrum of diseases called ciliopathies. Ciliopathies can affect a variety of organs due to the ubiquitous presence of primary cilia in different organ systems and their role as a signaling hub (Lancaster and Gleeson, 2009). The mechanosensory function of primary cilia occurs in various organs, such as renal nephron (Nauli et al., 2006, Siroky et al., 2006, Liu et al., 2005), hepatic biliary system (Masyuk et al., 2006), pancreatic duct(Cano et al., 2006) and vasculature(AbouAlaiwi et al., 2011, AbouAlaiwi et al., 2009). Shortened primary cilia have been shown to result in polycystic kidneys (Lin et al., 2003, Yoder et al., 1995), and a reduction in mechanosensory function of the cilia has also been reported to promote polycystic kidney phenotypes (Aboualaiwi et al., 2014, Nauli et al., 2008, Nauli et al., 2003). The ability to sense and respond to extracellular simulation is thought to be the basis for homeostatic adaptation and critical for normal development of various organs. When the primary cilium is dysfunction, cells cannot respond properly to environmental cues leading to ciliopathy phenotypes.

The primary cilium responds to flow-induced bending with calcium entry through mechanically sensitive channels. Studies have shown that bending of the cilium in cultured cells results in an increase in intracellular calcium concentration (Jin et al., 2014, Praetorius and Spring, 2001). The large increase in cytosolic calcium may activate calcium dependent processes that range from cell proliferation to cell death(Berridge et al., 2000).For this reason, calcium fluorimetry has been used as a means of quantifying cilia function, i.e. response to fluid-shear stress (Liu et al., 2005, Siroky et al., 2006)and pharmacological agents(Kathem et al., 2014, Abdul-Majeed and Nauli, 2011).

Polycystic kidney disease (PKD) has been associated to the inability of renal epithelia(Xu et al., 2007) or vascular endothelia(Nauli et al., 2008) to carry out a calcium influx in response to fluid-flow. Aberrant activation of mTOR (mammalian target of rapamycin) has also been reported in PKD(Shillingford et al., 2010, Shillingford et al., 2006, Pema et al., 2016). The loss of function in the TSC complex has been associated with development of renal cysts (Armour et al., 2012, Bonnet et al., 2009). In normal cells, the cilia regulate the mTOR pathway through polycystin-1 (PC1) mediated suppression. PC1 is localized to the cilia and is critical in the formation of the polycystin flow-sensing complex in the cilia. When PC1 is inactivated, cyst-lining epithelial cells show activation of mTOR as measured by phosphorylation of mTOR and S6 kinase, a downstream effector. This has led to the use of mTOR inhibitors, such as rapamycin, as potential drug candidate to target the aberrant mTOR activation in PKD.

Although the role of rapamycin on cilia length and function has never been studied, mTOR pathway may modulate cilia length and function, pointing to a potential effect of rapamycin on cilia length and function(Yuan et al., 2012). The relationship between mTOR and cilia also suggests that primary cilia are dynamic organelles that can be modulated through the mTOR intracellular signaling pathways. Because *in vivo* evidence has shown that rapamycin can reduce cyst growth and preserve renal function (Stayner et al., 2012, Shillingford et al., 2010, Tao et al., 2005), our present study aims to examine the effects of rapamycin-induced changes in cilia length and function. Our studies further evaluate if rapamycin would have an effect in vascular system in addition to the kidney. The use rapamycin also offers positive outcomes in different species, including mouse, rat, and pig(Annes et al., 2012). This indicates that them TOR pathway has a cross-species conserved element. To test this possibility of rapamycin effect on primary cilia, we use both pig and rodent cultured cells. Therefore, our hypothesis is that rapamycin will alter cilia length and function in porcine renal epithelial and mouse vascular endothelial cells.

## Materials and Methods

### Cell culture

Porcine renal epithelial cells from proximal tubule (LLCPK) and mouse vascular endothelial (ET) cells were cultured to a confluent monolayer in Dulbecco's Modified Eagle Medium (DMEM) supplemented with 10% fetal bovine serum (FBS) at 37°C in 5% CO2. For our cell proliferation studies, mouse inner medullary collecting duct (mIMCD) cells were cultured using the same conditions as mentioned above. The generations of LLCLPK, ET and mIMCD cells have been previously described (Hull et al., 1976, Nauli et al., 2008, Rauchman et al., 1993).Once confluent, cultured cells were incubated with media containing 2% FBS. We used less serum at this time to further induce cell differentiation. Fully differentiated cells tend to have optimal cilia length, and changes in cilia length can be measured more consistently. In some cases, the media contained different concentrations of rapamycin (0.01, 0.1, 1 and 10 μM) for 20 hours. For control experiments, vehicle alone was added to cells in the same manner and volume.

### Cilia length analysis

Primary cilia consist of acetylated microtubule structures and were measured by direct immunofluorescence with anti-acetylated α-tubulin staining in the absence or presence of 20-hour incubation with 1μM rapamycin. The cells were fixed for 10 minutes (4% paraformaldehyde/2% sucrose in PBS) and permeabilized for 5 minutes (10% triton X-100). Acetylated α-tubulin (1:10,000 dilution, Sigma Aldrich, St. Louis, MO) and fluorescein isothiocyanate (FITC)-conjugated (1:1000 dilution, Vector Labs Burlingame, CA) antibodies were each incubated with the cells for 1 hour at 37°C. Slides were then mounted with DAPI (Southern Biotech, Birmingham, AL) hard set mounting media. Nikon Eclipse Ti-E inverted microscope with NIS-Elements imaging software (version 4.30) was used to capture images of primary cilia. Automated image acquisition was conducted in 100X magnification fields and Z-stacks (0.1 μm slices) to create a large 3D image. This was done to select cilia from a confluent monolayer since it mimics the physiological structure of the epithelial and endothelial cells.

### Cytosolic calcium analysis

Cells were grown on glass-bottom plates to enable live microscopy imaging. After incubation for 20-hour without or with 1μM rapamycin, cells were loaded with 5 μM Fura2-AM (TEFLabs, Austin, TX) at 37°C for 30 min. Cells were then washed with DPBS (Dulbecco's Phosphate-Buffered Saline) and observed under a 40× objective lens with a Nikon Eclipse Ti-E microscope controlled by Elements software. Cytosolic calcium was observed by recording calcium-bound Fura excitation fluorescence at 340/380 nm and emission at 510 nm. Baseline calcium was observed for 2 minutes prior to data acquisition. Fluid-shear stress was then applied to cells utilizing an Instech P720 peristaltic pump with an inlet and outlet setup. The fluid was perfused on the glass-bottom plates at shear stress of 0.2, 0.6 or 1.0 dyne/cm^2^ for epithelial cells and 2.0, 5.0 or 8.0 dyne/cm^2^ for endothelial cells. After each experiment, the maximum calcium signal was obtained by perfusion of ATP (10μM) to confirm cell viability. Conditions for all experiments were maintained at 37°C and 5% CO_2_ in a stage top cage incubator (okoLab, Burlingame, CA). Calcium analysis was then followed a standard calculation as previously described (Upadhyay et al., 2014).

### Cell cycle analysis

To investigate a possibility of rapamycin effect on cell growth, cells were harvested with and without 1 μM rapamycin treatment. Cells were then fixed using 70% ethanol and incubated with propidium iodide (PI, 50 μg/ml), a DNA- intercalating fluorescent molecule, for 30 min at 37°C. To investigate cell cycle more accurately, cells were synchronized by physical separations based on their size and density. Cells were then grow for 36 hours and incubated with 10 μMBrdU (Invitrogen, Eugene, OR) for 1 hour at 37°C and 5% CO_2_ . Cells were trypsinized and fixed with 70% ethanol overnight at −20°C. After fixation the cells were permeabilized with 0.5% triton X-100 in PBS for 10 minutes. To denature DNA, a solution of 4N HCl with 1.0 % triton X-100 in ddH_2_O was used to incubate the cells for 20 minutes at room temperature followed by a quick neutralization step using 0.1M sodium borate, pH 8.5. The cells were then treated with Alexa 488 conjugated BrdU antibody (Invitrogen, Eugene, OR) at 1:25 dilution in PBS for 1 hour at at 37°C. Samples were stored in the dark for the antibody incubation and all steps afterwards. The cells were then stained with 50 μg/ml PI for 30 min at 37°C. Cell analysis was carried out with flow cytometry BDFacsverse with BD FAC suite software.

### Statistical analysis

Cilia length measurement consisted of n = 50–70 for each slide preparation, and a total of 4 slides was used in each group. Cytosolic calcium measurements consisted of n = 50 for each plate, and a total of 4 plates were used in each group. Cell cycle analysis consisted of n = 4 and a sample size of 10,000 for each individual experiment. All data are reported as mean ± standard error of mean with statistical power greater than 0.8 at p < 0.05. Data were analyzed utilizing ANOVA test followed by Tukey post-test for multiple groups. Analysis of data was performed with Prism GraphPad 5 software.

## Results

**Figure 1:**
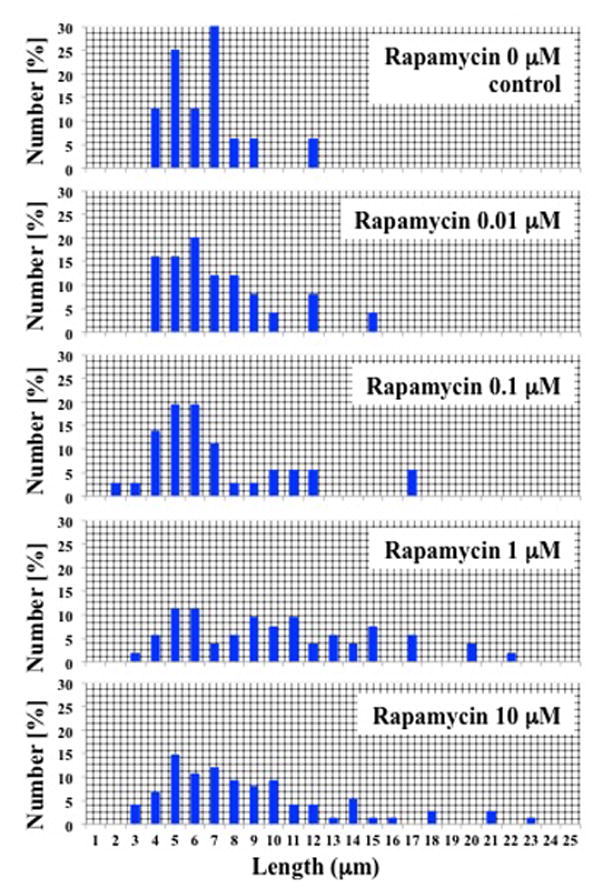
Aninitial screening indicates that rapamycin might increase the length primary cilia length in renal epithelial cells Cells were treated with various concentrations of rapamycin (0 to 10 mM). Length measurements were made from images taken at one single plane in triplicate. Cilia length was grouped in a discrete range, and percent distribution was tabulated for each rapamycin concentration.

**Figure 2:**
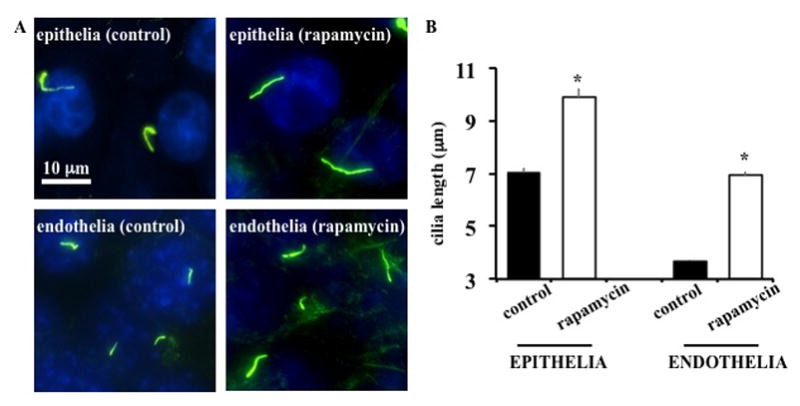
Treatment with rapamycin (1μM) increases primary cilia length in epithelial and endothelial cells **(A)** Cells were stained with ciliary marker acetylated-α-tubulin (green) and a nuclear marker (DAPI; blue). Rapamycin treatment increased cilia length in both cell lines. Each image was compiled from different z-stack captures. **(B)** Cilia length was significantly longer in rapamycin-treated cells, with a two-fold increase observed in endothelial cells. N=50–70 for each slide preparation, and a total of 4 slides was used in each group. * = p<0.05 compared to corresponding controls.

To find concentration of rapamycin that might affect the length of primary cilia, we carried out initial screening of rapamycin at a range of 0 mM to 10 mM in renal epithelial cells. Cilia length analysis using immunoflorescent-staining of acetylated α-tubulin shows the changes in cilia length distribution with different rapamycin concentrations (Figure 1). Treatment with rapamycin at a concentration of 1.0 μM gave an optimal and consistent percentage of cilia with longer lengths. Concentration of 1.0 μM was therefore selected for the rest of our experiments. We next confirmed the effect of rapamycin by acquiring the images three-dimensionally for a more precise measurement to account for cilia that appear at different focal planes (Supplemental Movie). In renal epithelial cells, average cilia length was 7.05 ± 0.15 μm. When treated with rapamycin (1.0 μM) for 20 hours, cilia length increased to 9.90 ± 0.33 μm showing an increase of almost 3.0 μm. In vascular endothelial cells the effect of rapamycin on cilia length was more pronounced. Compared to an average cilia length of 3.67 ± 0.04 μm in control endothelial cells rapamycin treatment increased cilia length to 6.94 ± 0.16 μm, almost twice the length of normal cilia. Statistical analysis showed significant differences in cilia length between the control vs. rapamycin-treatment in epithelial and endothelial cells (Figure 2).

**Figure 3:**
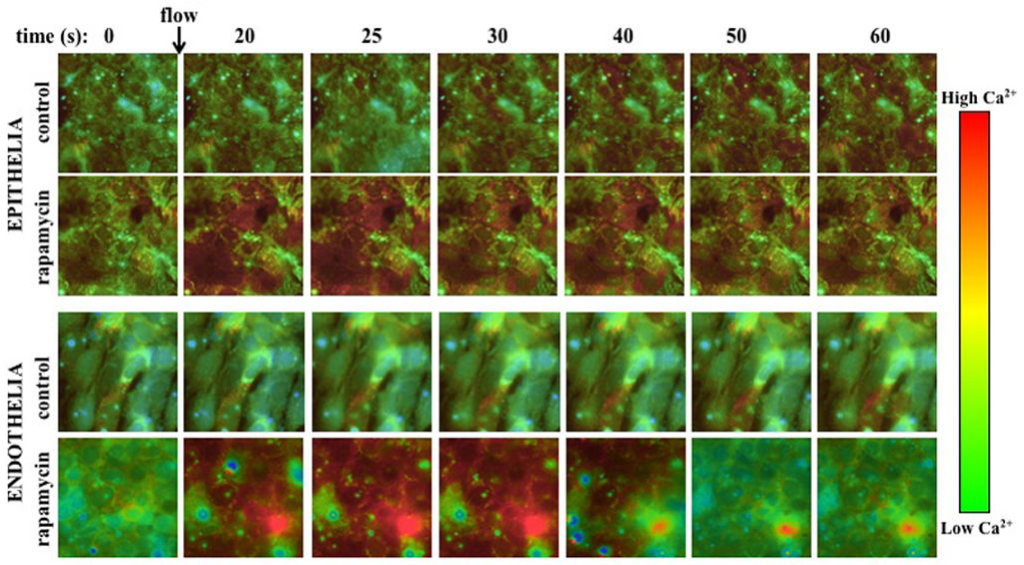
Fluid-shear stress induces calcium influx Representative Fura images at different time points from each group are shown, and the arrow indicates the start of fluid flow. Color bars indicate cytosolic calcium levels from low (green) to high (high).

**Figure 4:**
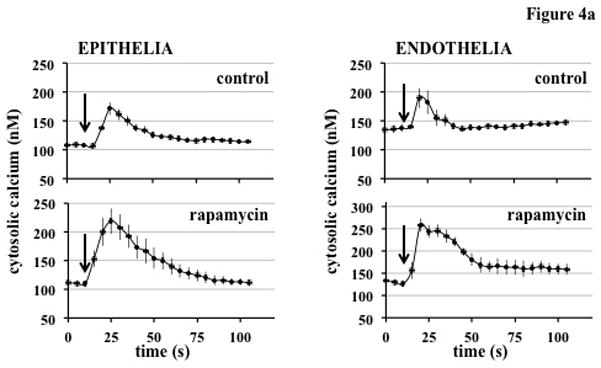

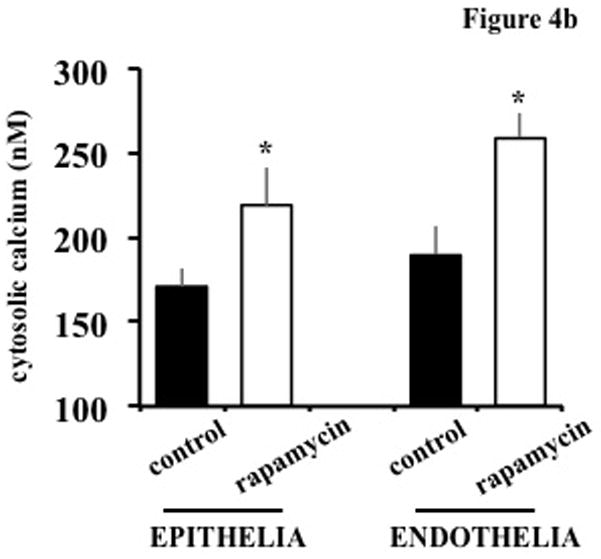
Flow-induced calcium influx into the cytoplasm is increased in cells treated with rapamycin **(A)** Intracellular calcium was measured in response to fluid-shear stress. The arrows indicate start of fluid flow. N=50 cells for each preparation, and a total of 4 preparations was used in each group. **(B)** Cilia function is assessed as peak of calcium influx in response to fluid-shear stress. Average peak calcium levels in control and rapamycin-treated cells are shown. Calcium peak is used to determine cilia function in response to fluid-shear stress. Cilia function is significantly greater inrapamycin-treated than in control cells. N=50 cells for each preparation, and a total of 4 preparations was used in each group. * = p<0.05 compared to corresponding controls.

As previous studies have shown, primary cilia responding to fluid flow can be observed through an influx of extracellular calcium (Siroky et al., 2006, Liu et al., 2005). For live-cell acquisition during flow experiments, cytosolic calcium level was measured with fura-2-AM a cell permeant calcium-specific indicator. After baseline measurement, cells were subjected to their optimal shear-stress and fura-2 fluorescence was captured at 510 nm. We observed an increase in the cytosolic calcium levels, which can be seen in representative pseudocolored images which correlate to cytosolic calcium levels (Figure 3). Our data show that rapamycin treatment enhances calcium influx after induction of shear stress in epithelial and endothelial cells (Figure 4A). Comparisons of peak calcium levels between control and rapamycin treated cells show significant increase in the maximum levels of calcium that is achieved upon exposure to shear stress (Figure 4B).

**Figure 5:**
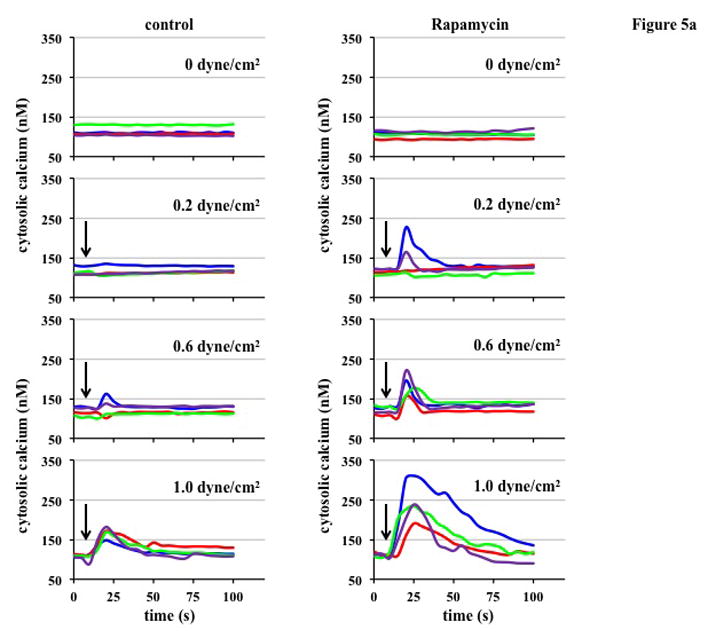

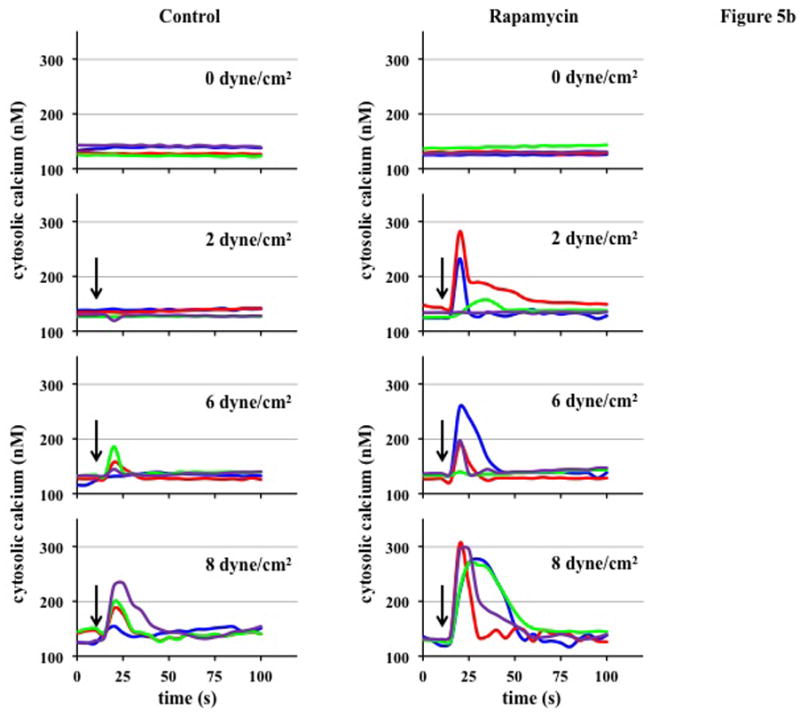

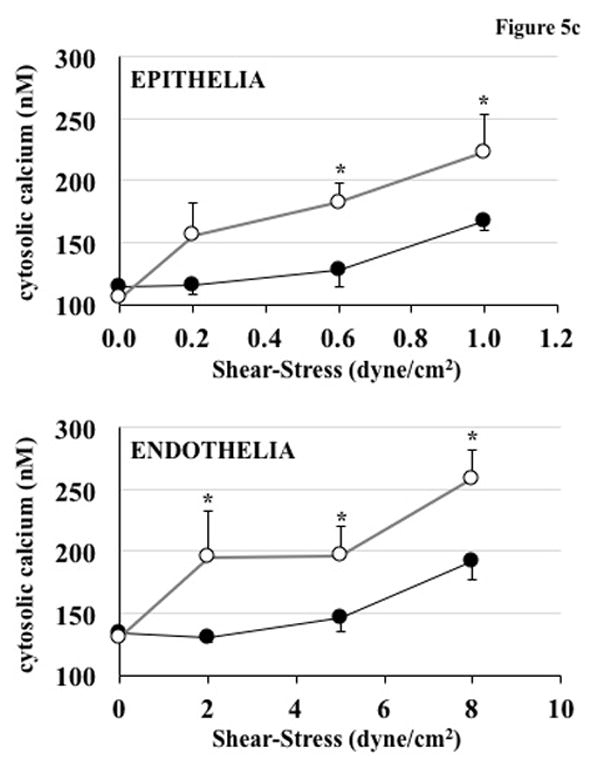
Rapamycin-treated cells show higher calcium influx in response to increasing various shear-stress forces In the time-lapse graph, baseline calcium levels were measured, and shear stress was applied at ~10 seconds (arrows). For epithelial cells a physiologically relevant range of 0–1.0 dyne/cm^2^ was used and for endothelial cells which experience higher ranges maximum shear stress used was 8 dyne/cm^2^ . (**A**) In renal epithelial cells, rapamycin treated cells showed higher levels of peak calcium in response to shear stress of varying magnitude. A shear stress of 0.2 dyne/cm^2^ was enough to cause calcium influx in rapamycin-treated cells. (**B**) Same effect was observed in vascular endothelial cells where rapamycin treatment increased the shear stress-induced calcium influx compared to control cells. (**C**) Peak calcium levels are shown on the graph at different levels of shear stress. In epithelial cells, significant increase in calcium influx was observed at shear stress levels ≥0.6 dyne/cm^2^ in rapamycin-treated vs. control cells. Endothelial cells also show significant increase in calcium influx when exposed to different levels of shear stress. For each calcium measurement, and a total of 4 experiments with N=50 for each was used. * = p<0.05 compared to corresponding controls.

Previous studies in our laboratories have indicated a correlation between cilia length and its ability to sense shear stress (Abdul-Majeed et al., 2012, Upadhyay et al., 2014). To examine if increase in cilia length from the rapamycin-treated cells would result in a greater sensitivity to fluid shear-stress, we used lower levels of shear stress and measured intracellular calcium influx in response to the simulation. In our epithelial cells we chose a physiologically relevant range of shear stress from 0.2 to a maximum of 1.0 dyne/cm^2^ (Figure 5A). In our control cells we observe a calcium response starting at shear-stress levels ≥ 0.6 dyne/cm^2^ . At the same level of shear-stress, rapamycin treated epithelial cells show a much higher influx of calcium and the calcium response is even present in a low shear-stress of 0.2 dyne/cm^2^ which is not seen in control cells. However, the trend of increased shear-stress inducing higher cytosolic calcium is seen in control cells, consistent with the idea that cells respond better to an optimal shear-stress level (Doerr et al., 2016, Nauli et al., 2008). This trend is also seen in endothelial cells, which line the vasculature and are exposed to higher levels of shear stress *in vivo* (Figure 5B). In our experiments with endothelial cells we chose to observe the effect of physiologically relevant range of shear stress starting from 2 dyne/cm^2^ to a maximum of 8 dyne/cm^2^. Rapamycin treated cells were able to initiate a calcium response at shear stress ≥ 2.0 dyne/cm^2^ while control cells only responded at shear stress ≥ 6.0 dyne/cm^2^ . Even the cytosolic calcium increase was reduced in control cells at different shear stress compared to rapamycin treated cells. Significant differences were observed between the shear stress-induced calcium influx in both epithelial and endothelial cells when compared to their respective control at varying levels of shear stress (Figure 5C).

**Figure 6:**
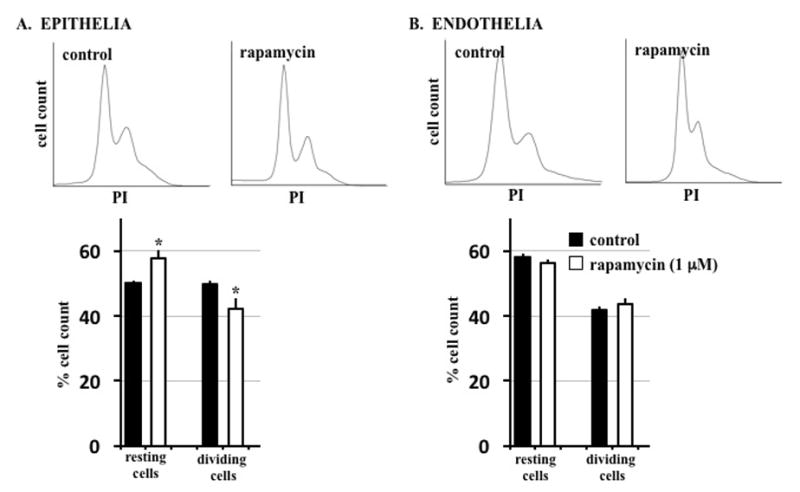
Treatment with rapamycin shows a minimal effect on the cell growth Cells were stained with propidium iodide (PI), a DNA intercalating agent, for flow cytometry analysis. **(A)** Epithelial cells showed a slight but significant increase in cells at the resting (G0) stage and lower percentage of cells undergoing mitosis when treated with rapamycin. **(B)** For endothelial cells, no significant variation in cell cycle was seen in rapamycin-treated cells compared to control. *= p<0.05 compared to corresponding controls.

Different models have been proposed for mTOR as a central regulator of cell metabolism, growth, proliferation and survival. Studies have shown that inhibition of the mTOR pathway using rapamycin leads to a G0/G1-phase arrest in yeast cells and mammalian lymphocytes(Fingar et al., 2004). It is widely known that cilia formation and elongation occurs during the resting phase. The same effect can be achieved by serum starvation, which results in cilia growing to their full extent(Nauli et al., 2013). With rapamycin known to be an anti-proliferative agent, we next examined the effect of rapamycin treatment on the cell growth distribution and whether growth arrest played a role in ciliary length increase. Significant differences were observed only in epithelial cells, where a higher percentage of cells were non-dividing compared to control (Figure 6). This same effect was not observed in endothelial cells and might be attributed to the different in species (pig vs. mouse) or cell types (renal epithelia vs. vascular endothelia).

**Figure 7:**
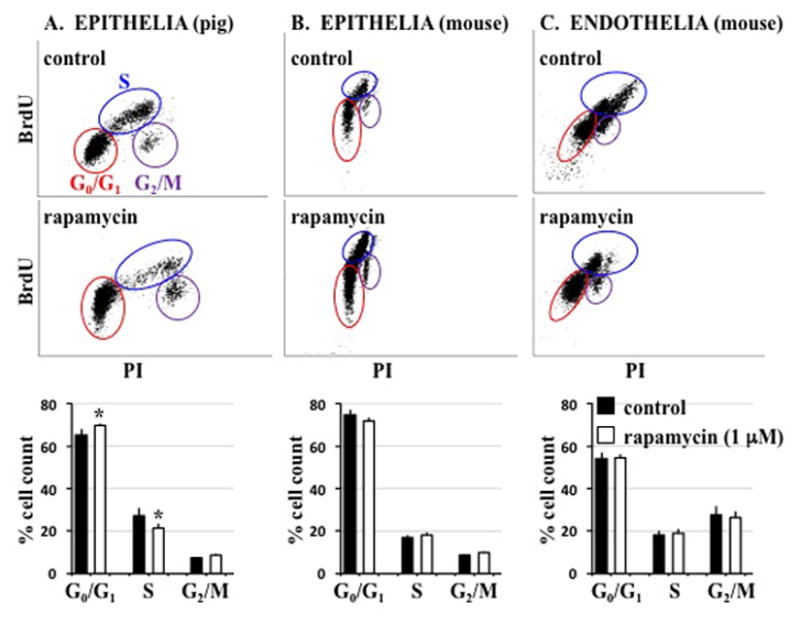
Treatment with rapamycin causes G1 phase arrest in porcine cells but not in mouse-derived cells In our cell cycle assay, BrdU incorporation was used to resolve cell cycle phases. **(A)** In epithelial cells treated with rapamycin the cell population was shifted more towards a G0/G1 phase by a significant amount. The rapamycin treated cells also had lower levels of BrdU incorporation indicating a G1 phase arrest. The population of cells in S-phase were found to be significantly reduced after rapamycin treatment. For mice epithelial **(B)** and endothelial **(C)** cells, no significant variation in cell cycle was seen in rapamycin-treated cells compared to control. * = p<0.05 compared to corresponding controls.

To investigate the possibility of species or cell type contribution on the differential effect of rapamycin, we included mouse renal epithelial cells in our study. BrdU was used to more specifically label dividing cells, and it was coupled with propidium iodide to stain total DNA in each cell. Consistent with previous study, we observed significant effect of rapamycin in cell cycle. However, the effect of rapamycin was only seen in porcine renal epithelial cells (Figure 7A) and not in mouse renal epithelial cells (Figure 7B) or mouse vascular endothelial cells (Figure 7C). A previous study has shown that inhibition of discrete mTOR downstream effectors may also be dependent on rapamycin concentration. The report suggests that a higher dose in the micromolar range of rapamycin was needed to completely block G1 cell cycle progression and that too was dependent on the sensitivity of the cells to rapamycin (Chatterjee et al., 2015). Thus, a potential effect of rapamycin to reduce cell division in endothelial cells remains possible at a higher concentration.

## Discussion

**Figure 8:**
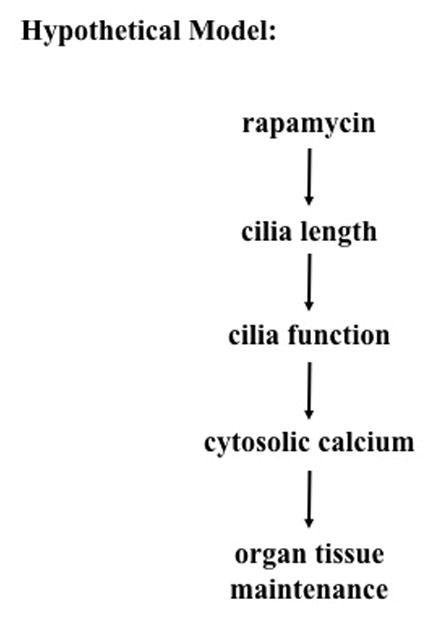
Hypothetical working model of rapamycin on cilia length and function Rapamycin increases cell sensitivity in response to fluid-shear stress. A greater sensitivity or response to shear stress is postulated to be a result of longer primary cilia. This, in turn, increases cilia function as denoted by an increase in shear-induced cytosolic calcium flux. Optimal length and function of primary cilia are thought to be required for proper architecture of tissue maintainane.

Primary cilia are critical sensory organelles that respond to flow in lumen-lining cells by initiating calcium influx into the cytoplasm. Using cytosolic calcium as a readout of ciliary function Upadhyay *et. al.* were able to show that cilia sensitivity to fluid-shear stress correlates with the length of cilia (Upadhyay et al., 2014). Our current studies indicate that rapamycin increases cilia length and enhances cilia sensitivity. We therefore hypothesize that rapamycin might increase cell sensitivity in response to fluid-shear stress through cytosolic calcium increase (as a readout) by enhancing cilia length and function. This, in turn, might have clinical relevant in preserving organ functions (Figure 8). In particular, many rodent models with no or short cilia length result in ciliopathy phenotypes, including cystic kidney, developmental delay, intellectual disability, and many others (Wallmeier et al., 2016, Ha et al., 2016, Lu et al., 2016, Zhang et al., 2011).Because cilia formation and function have been associated with cell-cycle(AbouAlaiwi et al., 2011, Plotnikova et al., 2009), we also investigated the roles of rapamycin in cell division. Our studies indicate that the effect of rapamycin on cell cycle may be species-dependent, although we cannot rule out that different species may have different sensitivity toward rapamycin.

Novel insights provided by *in vitro* studies and animal models may be utilized into designing treatments that improve ciliary structure/function. In the case of PKD, treatment strategies have included drugs that target disrupted mechanisms such as intracellular calcium, cAMP, CFTR chloride channels, or mTOR signaling(Torres, 2010, Yang et al., 2008, Magenheimer et al., 2006). Previous studies show that the mTOR pathway is aberrantly activated in PKD cells (Pei, 2010, Shillingford et al., 2006, Shillingford et al., 2010).mTOR is a serine/threonine kinase that provides the catalytic subunit for two distinct multi-protein complexes, mTORC1 and mTORC2. mTORC1 acts as metabolic sensor and is regulated by availability of amino acids, growth factors and energy stores. Activation of mTORC1 promotes both cell growth and proliferation. mTORC2 has been shown to function as an important regulator of the cytoskeleton organization, cell survival and polarity (Lieberthal and Levine, 2012, Loewith et al., 2002). Western blot analysis of orthologous animal models and humans with PKD show increased activation, i.e. phosphorylation of mTORC1 downstream targets and the 4E-BPs(Zafar et al., 2010). Rapamycin binds to FKBP12 and can inhibit both mTORC1 and, to a lesser extent, mTORC2 signaling(Zeng et al., 2007, Sarbassov et al., 2006).Rapamycin may induce glomerulonephritis and intratubular cast formation in protein overload nephropathy (Coombes et al., 2005, Marti and Frey, 2005). Nonetheless, treatment with rapamycin slows down cyst progression and improves renal function in animal models of PKD(Shillingford et al., 2006, Tao et al., 2005, Wahl et al., 2006, Zafar et al., 2010).Although the effect of reducing cyst burden by rapamycin has not been consistent in all animal models or in trials with PKD patients, substantial evidence linking aberrant mTOR activity to cystogenesis suggests that mTOR might be one of complex intertwined pathways in the pathogenesis of PKD and other ciliopathies.

We use both porcine and mouse cells in the present studies. The porcine cells were generated previously and have been used because humans are more closely related to pigs than to rodents, particularly with regard to renal physiology (Heussner and Dietrich, 2013, Hull et al., 1976).These porcine cells also display inherent characteristics of structural organization and transepithelial transport functions similar to those renal epithelia observed under *in vivo* conditions within the proximal tubules. Thus, it is thought that these porcine cells might represent an alternative to human primary cells. The mouse cells were generated previously from aorta and retain its vascular properties and endothelial markers (AbouAlaiwi et al., 2011, Brookes et al., 2013, Nauli et al., 2008). The functional studies of these cells have also shown that these cells retain cellular responses to various mechanical forces and pharmacological agents. In addition, these cells have normal directional migration, monolayer permeability and cytoskeletal organization(Jones et al., 2012).

Endothelial cells, which line the inner lumen of blood vessels, depend on cilia to sense the shear stress. Observations of vascular endothelial cells lacking cilia show that without functional cilia, there is no calcium influx in response to fluid flow (Nauli et al., 2008). This prevents the vasculature to adapt with variable blood flow levels since the bending of primary cilia also regulates the production of nitric oxide (NO), an important vasodilator(Nauli et al., 2008). Abnormalities in cilia function have been associated with defective NO production resulting in hypertension (Rahbari-Oskoui et al., 2014), vascular aneurysm (Aboualaiwi et al., 2014) in addition to cyst formation in the kidneys(Ecder and Schrier, 2009).

In our study, we observed that rapamycin has an effect in the cilia of porcine epithelial and mouse endothelial cells. The results show that in addition to increasing cilia length, rapamycin-treated cells showed a higher cytosolic calcium response when challenged to fluid flow. This is in agreement with previous studies done with dopaminergic agents that enhanced cilia length and function(Upadhyay et al., 2014).Typically, ciliogenesis occurs during the G0/G1 phase when the centriole becomes the basal body that serves as a foundation for attachment of ciliary subunits. With the help of interflagellar transport (IFT) proteins the cilia keeps elongating till it reaches its determined length. This induction of optimum ciliary length can be observed in cilia of cells subjected to serum deprivation to induce a G0/G1 arrest. Because rapamycin is an antiproliferative agent, we did cell cycle analysis to determine if our concentration of 1 μM rapamycin caused any changes to the cell cycle distribution, which could have affected our ciliary length observations. Our FACS results show than only porcine epithelial cells show a slight increase population of cells in the G0/G1 phase. To examine whether this was a cell line specific or species-specific response we used mouse renal epithelia and observed no significant changes in cell cycle distribution.

In healthy renal epithelia or vascular endothelia, primary cilia bend when exposed to physiological flow and initiate an increase in cytosolic calcium in addition to initiating modulation of other signaling pathways important for normal tissue homeostasis. Using influx of extracellular calcium as a marker of cilia function in response to fluid flow, we were able to observe an increase in ciliary function with rapamycin treatment.

Rapamycin has been effective in decreasing cyst progression but its effect on the cilia structure/function has not received much attention. Primary cilia have been established as important flow sensing organelles and shortened cilia fail to translate the mechanical stimulation to downstream intracellular responses. Recently studies showed that primary cilia can regulate mTOR activity(Aznar and Billaud, 2010, Boehlke et al., 2010). MDCK cells grown under permanent fluid flow for several days showed decreased mTOR activity compared to cilia-less Kif3a knockdown cells. This *in vitro* study demonstrated that flow-induced bending of the cilium activates Lkb1-mediated phosphorylation of AMPK at the basal body of the cilia, inhibiting mTORC1 activity and regulating cell size (Boehlke et al., 2010). The ability of cilia to silence mTOR and the reciprocal increase in cilia length by mTOR inhibition indicate that the cilia-mTOR pathway may have much more in common than previously thought. Although the rationale of rapamycin treatment for PKD or TSC hinges on the aberrant mTOR pathway, our study shows that rapamycin plays a role in cilia length/function providing tentative evidence that rapamycin could potentially be used as a ciliotherapy. In healthy cells there is a defined range of cilia length and it is dependent on the shear stress experienced by the system. In places with high levels of shear stress, such as the vasculature, cilia are usually shorter. In the renal nephron where the filtrate flows comparatively slowly, the epithelial cells have longer cilia that can extend outward into the lumen to sense flow, which is unhindered by the drag forces near the wall.

Our studies offer a fundamental idea that rapamycin has an effect on primary cilia. Whether effect of rapamycin on cilia is direct or indirect, future studies are warranted for a better in-depth understanding to extrapolate the results from animal cells into clinical practice. Importantly, our results require further research to understand the pathways connecting the primary cilium, mTOR signaling and cystogenesis in ciliopathies.

## Supplementary Material

Supp_Movie Supplemental Movie Shown here is a representative approach of our data analysis on cilia length. To enable a more accurate measurement of cilia, the images of primary cilia (green) and nucleus (blue) were taken three-dimensionally. This allows measurement of cilia that are within a single focal plane and at a different focal plane.
